# The Design of Diatomite/TiO_2_/MoS_2_/Nitrogen-Doped Carbon Nanofiber Composite Separators for Lithium–Sulfur Batteries

**DOI:** 10.3390/ma18153654

**Published:** 2025-08-04

**Authors:** Wei Zhong, Wenjie Xiao, Jianfei Liu, Chuxiao Yang, Sainan Liu, Zhenyang Cai

**Affiliations:** 1 School of Materials Science and Engineering, Central South University, Changsha 410083, China; zhong9340@gmail.com; 2 School of Minerals Processing and Bioengineering, Central South University, Changsha 410083, Chinacsu245601012@csu.edu.cn (J.L.);; 3 School of Business, Central South University, Changsha 410083, China; lucas04031@163.com

**Keywords:** lithium–sulfur batteries, molybdenum disulfide, carbon nanofiber, titanium dioxide, diatomite

## Abstract

Severe polysulfide shuttling and sluggish redox kinetics critically hinder lithium–sulfur (Li-S) battery commercialization. In this study, a multifunctional diatomite (DE)/TiO_2_/MoS_2_/N-doped carbon nanofiber (NCNF) composite separator was fabricated via hydrothermal synthesis, electrospinning, and carbonization. DE provides dual polysulfide suppression, encompassing microporous confinement and electrostatic repulsion. By integrating synergistic catalytic effects from TiO_2_ and MoS_2_ nanoparticles, which accelerate polysulfide conversion, and conductive NCNF networks, which facilitate rapid charge transfer, this hierarchical design achieves exceptional electrochemical performance: a 1245.6 mAh g^−1^ initial capacity at 0.5 C and 65.94% retention after 200 cycles. This work presents a rational multi-component engineering strategy to suppress shuttle effects in high-energy-density Li-S batteries.

## 1. Introduction

Lithium–sulfur (Li-S) batteries demonstrate considerable promise for use in various energy storage devices due to their exceptionally high theoretical energy density of up to 2600 Wh kg^−1^ [1,2]. Furthermore, sulfur, the cathode active material, offers significant advantages, including its natural abundance, environmental friendliness, and low cost [3,4,5]. However, commercialization of these batteries is hindered by the polysulfide shuttle effect and sluggish redox kinetics. Soluble LiPS intermediates dissolve into the electrolyte, causing active material loss and rapid capacity decay [6,7]. Moreover, slow LiPS conversion kinetics reduce sulfur utilization and accelerate battery degradation [8]. Critically, LiPSs diffusing to the anode undergo parasitic reactions with lithium metal, forming insoluble Li_2_S_2_/Li_2_S deposits on its surface [9]. These intertwined issues, particularly the shuttle effect and sluggish kinetics, represent a key bottleneck for Li-S battery development [10].

Developing efficient electrocatalytic host materials is a key strategy for overcoming these challenges. To date, various materials have been reported for constructing Li-S battery cathode hosts. For instance, metal oxides (such as TiO_2_ [11,12,13], ZnO [14], WO_3_ [15], MoO_3_ [16,17,18], etc.) typically exhibit strong adsorption capabilities towards LiPSs, effectively anchoring them. Among these oxides, TiO_2_ is considered a promising material owing to its excellent chemical stability and catalytic activity towards LiPSs. However, its catalytic activity remains insufficient for efficient LiPS conversion, a problem compounded by its inherently low electrical conductivity. In contrast, metal sulfides (ZnS [19], WS_2_ [20], FeS_2_ [21], etc.) generally possess superior catalytic capability and higher conductivity. Nevertheless, they exhibit weaker LiPS adsorption, making it challenging for single sulfides to achieve strong adsorption and efficient catalysis simultaneously. The two-dimensional layered structure of MoS_2_, bound by van der Waals forces, offers both chemical bonding sites (via polar groups) and physical confinement to suppress LiPS diffusion. However, MoS_2_ suffers from inherent drawbacks like poor electrical conductivity and aggregation tendency. These limitations lead to sluggish kinetics and inadequate catalytic activity [22], hindering its effectiveness against the shuttle effect and long-term capacity fading.

Therefore, designing composite hosts that integrate strong LiPS adsorption, high conductivity, and efficient catalysis is crucial. Carbon-based materials like carbon nanofibers (CNFs) are commonly used as conductive scaffolds due to their good conductivity and tunable morphology. However, pure carbon offers only limited physical LiPS adsorption and lacks catalytic sites. Introducing components with strong chemisorption and catalytic capabilities (e.g., metal oxides/sulfides) into the carbon matrix effectively addresses these limitations. Naturally abundant and low-cost diatomite (DE) is a promising candidate for suppressing the LiPS shuttle effect due to its unique properties. DE primarily comprises amorphous SiO2 with minor amounts of polar oxides (such as Al_2_O_3_ and Fe_2_O_3_) [23], all contributing to LiPS adsorption [24,25,26]. Crucially, its exceptionally rich natural microporosity, combined with abundant surface Si−OH groups, provides strong LiPS binding sites and enhances electrolyte wettability [27,28]. Hence, diatomite is a strong candidate for use as a functional filler for carbon-based composites.

Based on the above considerations, this study innovatively integrates diatomite (DE) with TiO_2_ and MoS_2_ into a nitrogen-doped carbon nanofiber (NCNF) matrix, designing and fabricating a DE/TiO_2_/MoS_2_/NCNF composite material. This composite was prepared via electrospinning followed by carbonization. Diatomite (DE) is a key component of the composite. Its rich microporous structure and surface Si−OH groups provide numerous adsorption and confinement sites for LiPSs within the three-dimensional TiO_2_/NCNF network while exerting electrostatic repulsion. Simultaneously, the continuous conductive network constructed by TiO_2_ nanoparticles and conductive carbon nanofibers effectively compensates for the inherent low conductivity of MoS_2_. More importantly, the interfacial synergy among DE, TiO_2_, MoS_2_, and the carbon fibers significantly promotes the adsorption, diffusion, and liquid–solid conversion processes of LiPS intermediates. Electrochemical performance tests demonstrate that Li-S batteries employing a DE/TiO_2_/MoS_2_/NCNF-coated separator achieve a high initial discharge specific capacity of 1245.6 mAh g^−1^ at 0.5 C. After 200 charge/discharge cycles, a capacity retention rate of 65.94% is maintained, with the remaining discharge specific capacity stabilizing at 821.3 mAh g^−1^, showcasing excellent cycling stability.

## 2. Experimental

### 2.1. Material Preparation

Diatomite (DE) pretreatment was performed using secondary diatomite ore from Changbai Mountain via a water-washing, acid-leaching, and water-washing purification process. Specifically, 10 g of raw diatomite ore was dispersed in 500 mL of deionized water, magnetically stirred for 12 h, and then allowed to settle for 12 h. The supernatant was discarded, and the lower sediment was collected and dried at 60 °C to obtain activated diatomite. Subsequently, 5 g of this activated diatomite was added to a 2 mol L^−1^ dilute sulfuric acid solution at a solid-to-liquid ratio of 1:10 (g/mL). The mixture was magnetically stirred in a 60 °C water bath for 3 h. After cooling to room temperature, the product was repeatedly centrifuged and washed with deionized water until the supernatant reached a neutral pH. Finally, the acid-washed precipitate was dispersed in 1000 mL of deionized water, magnetically stirred for 12 h, and allowed to settle for 12 h. The supernatant was discarded, and the lower sediment was collected, washed to neutrality, and dried at 60 °C to yield purified diatomite (DE).

The DE/MoS_2_ composite was synthesized via a hydrothermal method. Initially, 1.2358 g of ammonium molybdate tetrahydrate ((NH_4_)_6_Mo_7_O_24_·4H_2_O), 1.0656 g of thiourea (CH_4_N_2_S), and 0.2 g of purified DE were added to 30 mL of deionized water and magnetically stirred for 30 min to form a homogeneous dispersion. This solution was transferred to a Teflon-lined autoclave (Zhongshiyi Instrument Equipment Co., Ltd., Zhengzhou, China) and reacted at 200 °C for 24 h. After natural cooling to room temperature, the product was separated by centrifugation. The solid precipitate was alternately washed several times with absolute ethanol and deionized water, followed by vacuum drying at 60 °C for 24 h to obtain a dark gray DE/MoS_2_ composite powder. For comparison, pure MoS_2_ was synthesized under identical conditions without adding diatomite.

The diatomite/TiO_2_/MoS_2_/N-doped carbon nanofiber composite (DE/TiO_2_/MoS_2_/NCNF) was prepared by electrospinning combined with carbonization. The precursor solution was first prepared as follows: 6 mL of N,N-dimethylformamide (DMF), 1 mL of absolute ethanol, and 2 mL of glacial acetic acid were added to a 10 mL glass vial under magnetic stirring. Under continuous stirring, 2 mL of tetrabutyl titanate (Ti(OC_4_H_9_)_4_) was slowly added dropwise. Subsequently, 0.1 g of the DE/MoS_2_ composite, 0.4 g of urea (CO(NH_2_)_2_), and 1.125 g of polyvinylpyrrolidone (PVP, Mw ≈ 1,300,000) were sequentially added. The vial was sealed and magnetically stirred continuously at room temperature for 24 h to obtain a homogeneous spinning solution. For electrospinning, the solution was loaded into a 10 mL syringe fitted with a 22 G flat-tip metal needle (inner diameter: 0.6 mm). The syringe was mounted on the electrospinning apparatus. The feeding rate was set to 0.2 mm min^−1^, the needle tip-to-collector (drum) distance was 15 cm, and an applied voltage of 25 kV was used. Fibers were uniformly collected onto silicone oil-coated aluminum foil. The collected fiber mat was peeled off and dried at 60 °C for 2 h to remove residual solvent. The mat was then pre-oxidized in a muffle furnace under air atmosphere, being heated to 260 °C at 2 °C min^−1^ and held for 2 h. The pre-oxidized sample was transferred to a tube furnace and carbonized under argon atmosphere, heated to 800 °C at 5 °C min^−1^, held for 2 h, and naturally cooled to room temperature to yield the DE/TiO_2_/MoS_2_/NCNF composite. The control sample DE/MoS_2_/NCNF was prepared using the same precursor solution but omitting tetrabutyl titanate. The TiO_2_/MoS_2_/NCNF sample was prepared by replacing DE/MoS_2_ with an equal mass of pure MoS_2_ in the precursor solution.

### 2.2. Preparation of Composite-Coated Separators

The active material (DE/TiO_2_/MoS_2_/NCNF or control material), acetylene black (AB) conductive agent, and the polyvinylidene fluoride (PVDF) binder were precisely weighed and mixed at a mass ratio of 8:1:1. An appropriate amount of N-methyl-2-pyrrolidone (NMP) solvent was added, and the mixture was magnetically stirred for 6 h to form a homogeneous and stable slurry. This slurry was uniformly coated onto one side of a commercial polypropylene (PP) separator (Celgard 2500, Celgard, Charlotte, NC, USA) using a 100 μm doctor blade. The coated separator was dried in a vacuum oven at 60 °C for 12 h to completely remove the NMP solvent. The dried separator was cut into 16 mm diameter disks using a precision cutter (PinChuang Technology Development Co., Ltd., Tianjin, China). The areal density of the active material in the coating was controlled to be approximately 1.3 mg cm^−2^. All control separators (e.g., DE/MoS_2_/NCNF-coated, TiO_2_/MoS_2_/NCNF-coated) were prepared following identical procedures to ensure consistent experimental conditions.

### 2.3. Preparation of Li-S Battery Cathodes

Sublimed sulfur (S) and acetylene black (AB) were mixed at a mass ratio of 3:1 and thoroughly ground. The mixture was thermally treated at 155 °C for 12 h under argon atmosphere to allow sulfur melting and infiltration into the AB pores, resulting in a sulfur–carbon composite (AB@S). Subsequently, AB@S, an additional AB conductive agent, and a PVDF binder were mixed at a mass ratio of 8:1:1. An appropriate amount of NMP solvent was added, and the mixture was magnetically stirred to form a homogeneous slurry. The slurry was coated onto an aluminum foil current collector using a doctor blade. The coated electrode was dried in a vacuum oven at 60 °C for 12 h. Finally, the dried electrode was cut into 12 mm diameter disks as cathodes, with a sulfur areal loading controlled at approximately 1.3 mg cm^−2^.

### 2.4. Assembly of Li-S Coin Cells

All cell assembly steps were performed inside an argon-filled glove box (Vigor Technology Corporation, Suzhou, China) (H_2_O < 0.01 ppm, O_2_ < 1 ppm). The negative can of a CR2032 coin cell was placed on a clean surface. A stainless-steel spacer and wave spring (conductive side facing up) were sequentially placed inside the can. A lithium metal disk (diameter 15.6 mm) was placed on the spacer as the anode. An appropriate amount of electrolyte (1 M lithium bis(trifluoromethanesulfonyl)imide (LiTFSI) + 0.1 M lithium nitrate (LiNO_3_) in a 1:1 *v*/*v* mixture of 1,3-dioxolane (DOL) and dimethoxyethane (DME)) was dropped onto the lithium surface. The total volume of the electrolyte for a single battery is 25 μL. The electrolyte-to-sulfur (E/S) ratio used in this study was 17.0 μL mg^−1^. The prepared functionalized separator (diameter 16 mm) was placed on the lithium disk with the coating layer facing the cathode side. Additional electrolyte was added to ensure complete wetting of the separator. The prepared sulfur cathode disk (diameter 12 mm) was centered on the other side of the separator (i.e., on top of the coating), forming a “Li anode–functional separator–S cathode” sandwich structure. The positive can was placed on top, and the cell was crimped sealed using a coin cell crimper. Excess electrolyte was wiped from the cell casing with lint-free paper before removal.

### 2.5. Material Characterization and Electrochemical Testing

Material phase analysis was performed using a Rigaku DX-2500 X-ray diffractometer (XRD, Rigaku, Tokyo, Japan) with Cu Kα radiation (λ = 1.5418 Å). Scans were conducted from 10° to 80° (2θ) at a rate of 5° min^−1^. The chemical bonding structure was analyzed using a Thermo Scientific Nicolet iS50 Fourier transform infrared spectrometer (FTIR, Thermo Fisher Scientific, Waltham, MA, USA) over the range 400–4000 cm^−1^, with a resolution of 4 cm^−1^. Surface elemental composition and chemical states were analyzed using a Thermo Fisher Scientific K-Alpha X-ray photoelectron spectrometer (XPS, Thermo Fisher Scientific, Waltham, MA, USA) with an Al Kα X-ray source (1486.6 eV). Sample morphology and microstructure were observed using a TESCAN MIRA 3 LMH field-emission scanning electron microscope (SEM, TESCAN Group a.s., Brno, Czech Republic). Elemental mapping was performed using an Oxford Instruments Ultim Max 80 energy-dispersive X-ray spectroscopy (EDS) system (Oxford Instrument Technology (Shanghai) Co., Ltd., Shanghai, China). Microstructural analysis was conducted using a JEOL JEM-F200 field-emission transmission electron microscope (TEM, JEOL Ltd., Tokyo, Japan) operating at 200 kV.

Electrochemical performance was evaluated using a CHI 660E electrochemical workstation (Shanghai Chenhua Instrument Co., Ltd., Shanghai, China). Cyclic voltammetry (CV) measurements were performed within a voltage window of 1.7–2.8 V (vs. Li^+^/Li) at scan rates of 0.1, 0.2, 0.3, 0.4, and 0.5 mV s^−1^. Electrochemical impedance spectroscopy (EIS) was conducted at open-circuit potentials over a frequency range of 100 kHz to 10 mHz, with an AC perturbation amplitude of 5 mV. Impedance data were fitted using Zview v.40h software. Galvanostatic charge–discharge cycling tests were performed using a LAND CT2001A battery test system (Wuhan Landian Electronics Co., Ltd., Wuhan, China) at a current density of 0.5 C (1 C = 1675 mA g^−1^, based on sulfur mass) within a voltage window of 1.7–2.8 V to evaluate specific capacity, cycling stability, and rate capability.

## 3. Results and Discussion

**Figure 1:**
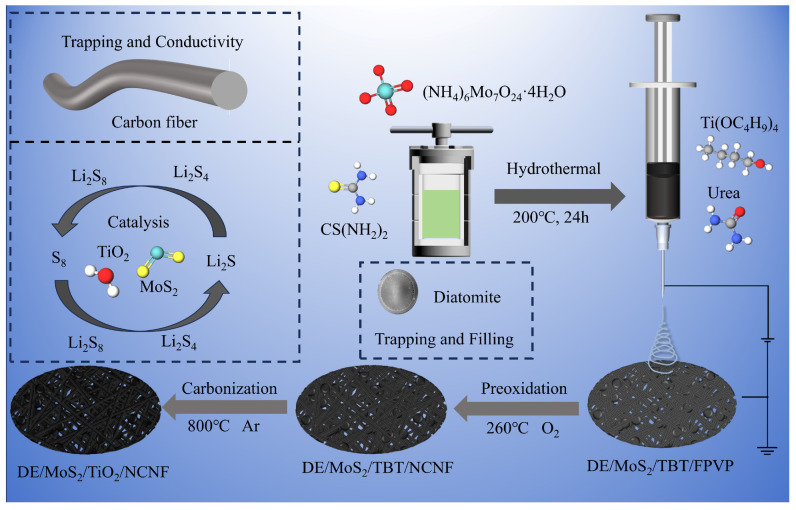
Schematic diagram of the preparation of the DE/TiO_2_/MoS_2_/NCNF composite material.

In this study, DE/MoS_2_ was synthesized by the conventional hydrothermal method. Subsequently, DE/TiO_2_/MoS_2_/NCNF composite functional materials were constructed by electrospinning and carbonization processes to modify the lithium–sulfur battery separator, as shown in Figure 1.

### 3.1. Phase Analysis

The DE/MoS_2_ composites were systematically characterized, to explore the phase characteristics. The XRD pattern (Figure S1a) shows that the characteristic peaks of amorphous SiO_2_ (2θ = 21.8°) and MoS_2_ (14.13°, 32.91°) are simultaneously present in the DE/MoS_2_ composite material, confirming the coexistence of the two phases. The infrared spectrum (Figure S1b) indicates that the composite material retains the Si-O-Si bond of diatomite (1100 cm^−1^) and the S-Mo-S vibration peak of MoS_2_ (1049 cm^−1^). XPS analysis (Figure S2) confirmed that Mo exists in the +4-valence state and S is coordinated in the form of S^2−^.

**Figure 2:**
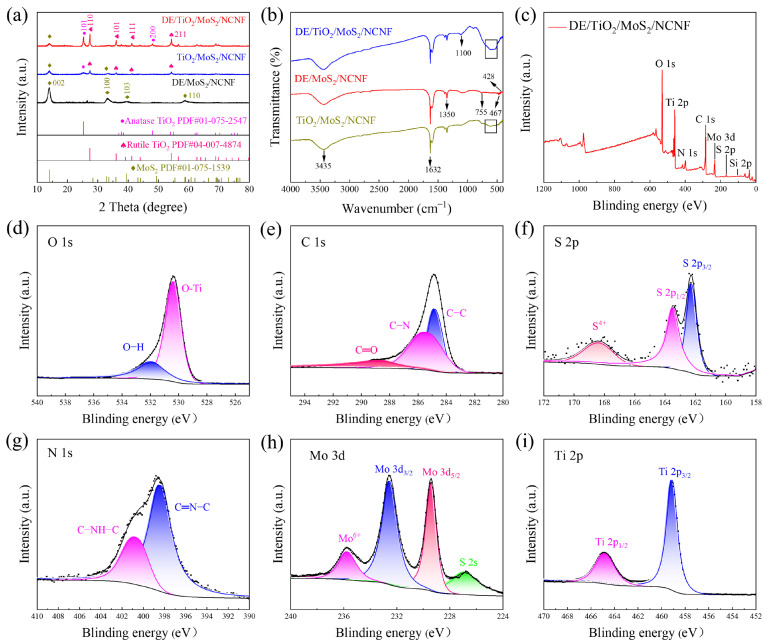
(**a**) XRD patterns of DE/MoS_2_/NCNF, DE/TiO_2_/MoS_2_/NCNF, and TiO_2_/MoS_2_/NCNF composites. (**b**) Infrared spectra of DE/MoS_2_/NCNF, DE/TiO_2_/MoS_2_/NCNF, and TiO_2_/MoS_2_/NCNF composites. (**c**) The XPS survey spectra of DE/TiO_2_/MoS_2_/NCNF composites. (**d**–**i**) High-resolution XPS spectra of O 1s, C 1s, S 2p, N 1s, Mo 3d, and Ti 2p (the black dots represent the raw data, and the black line is the fitted line.).

The crystalline phase structures of the prepared materials were analyzed using X-ray diffraction (XRD) patterns. Figure 2a shows the XRD patterns of the DE/MoS_2_/NCNF, TiO_2_/MoS_2_/NCNF, and DE/TiO_2_/MoS_2_/NCNF materials. For DE/MoS_2_/NCNF, diffraction peaks observed at 14.1°, 32.9°, and 39.5° correspond to the (002), (100), and (103) planes of MoS_2_ (PDF#01-075-1539), respectively. In the XRD patterns of TiO_2_/MoS_2_/NCNF and DE/TiO_2_/MoS_2_/NCNF, characteristic peaks of MoS_2_ were present, and additional peaks were observed at 25.3° and 48.0° [29], corresponding to the (101) and (200) planes of anatase TiO_2_, respectively. Peaks at 27.4°, 36.1°, 41.2°, and 54.3° correspond to the (110), (101), (111), and (211) planes of the rutile phase [30], respectively. These XRD results confirm the presence of both MoS_2_ and a composite phase of TiO_2_ crystals in the synthesized samples. Figure 2b shows the Fourier transform infrared (FTIR) spectra of DE/MoS_2_/NCNF, DE/TiO_2_/MoS_2_/NCNF, and TiO_2_/MoS_2_/NCNF. Absorption peaks near 3700–3050 cm^−1^ were observed in all spectra, corresponding to the stretching vibrations of silanol groups (Si-OH) in diatomite and hydroxyl groups of surface-bound water. The peak at 1632 cm^−1^ is attributed to the bending vibration of water hydroxyl groups (-OH) [31]. The peak near 1350 cm^−1^ corresponds to the C-N bond vibration. Peaks at 1100, 755, and 467 cm^−1^ correspond to the transverse, longitudinal symmetric stretching, and bending vibrations of the Si-O-Si bond [32], respectively. The peaks at 1049 cm^−1^ and 428 cm^−1^ are assigned to the bending vibration of S-Mo-S and the Mo-S stretching vibration [30], respectively, indicating that the DE/MoS_2_ composite retained the fundamental functional groups of both diatomite and MoS_2_. Furthermore, in the FTIR spectra of DE/TiO_2_/MoS_2_/NCNF and TiO_2_/MoS_2_/NCNF, the characteristic peaks of diatomite were weakened, and a broad absorption band appeared in the range of 500–700 cm^−1^, which is attributed to the Ti-O bonds of the doped TiO_2_ [33].

X-ray photoelectron spectroscopy (XPS) analysis revealed crucial information on chemical bonding states, elemental species, and electronic structures. The full survey scan (Figure 2c) confirmed the presence of C, O, N, Mo, Si, Ti, and S. High-resolution spectra (Figure 2d–i) were deconvoluted for the O 1s, C 1s, S 2p, Mo 3d, N 1s, and Si 2p orbitals as follows: The O 1s spectrum (Figure 2d) shows the existence of O-H and O-Ti bonds. The C 1s spectrum (Figure 2e) exhibited peaks at 284.8 eV, 285.8 eV, and 289.2 eV, assigned to C-C/C=C, C-N, and C=O functional groups, respectively, indicating nitrogen doping within the carbon matrix [34]. The S 2p spectrum (Figure 2f) showed peaks at 163.2 eV (S 2p_1/2_) and 162.1 eV (S 2p_3/2_), characteristic of MoS_2_ formation. Additionally, two small peaks near 169.0 eV suggest the presence of S^4+^ at MoS_2_ edges. The N 1s spectrum (Figure 2g) displayed peaks at 398.3 eV (pyridinic N) and 400.6 eV (graphitic N), confirming the nitrogen doping configurations. The Mo 3d spectrum [35,36] (Figure 2h) was deconvoluted into four peaks: the S 2s peak at 226.6 eV, Mo 3d_5/2_ at 229.4 eV, Mo 3d_3/2_ at 232.6 eV, and a Mo^6+^ peak at 235.7 eV, indicating slight surface oxidation of Mo^4+^. The Ti 2p spectrum [37] (Figure 2i) showed peaks at 465.1 eV (Ti 2p_1/2_) and 459.3 eV (Ti 2p_3/2_). Collectively, these results confirm the successful synthesis of the DE/TiO_2_/MoS_2_/NCNF composite.

### 3.2. Morphology and Structure Analysis

**Figure 3:**
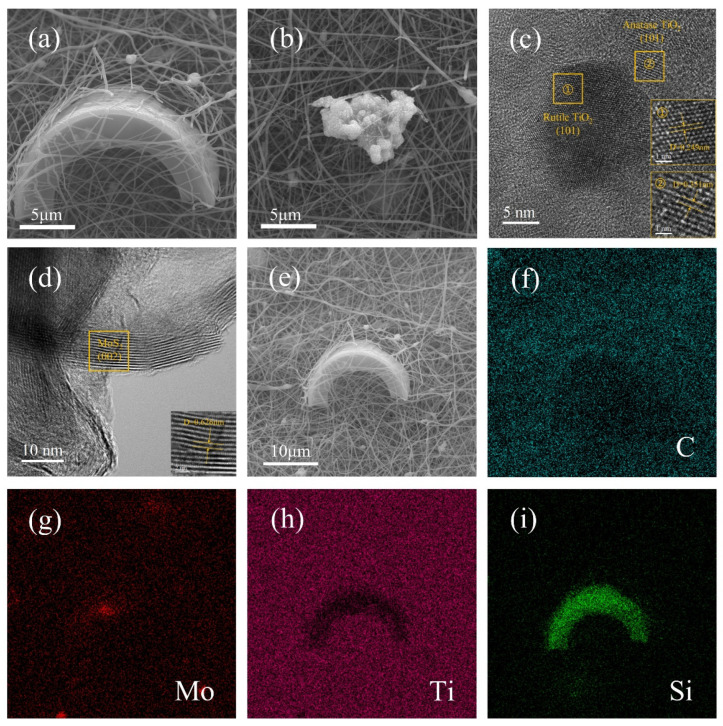
(**a**,**b**) SEM micrographs; (**c**,**d**) high-resolution TEM image; (**e**–**i**) elemental mapping of DE/TiO_2_/MoS_2_/NCNF materials.

To investigate the microstructure, interfacial bonding, and elemental distribution of the DE/MoS_2_ and DE/TiO_2_/MoS_2_/NCNF composite, comprehensive characterization was performed using scanning electron microscopy (SEM), high-resolution transmission electron microscopy (HR-TEM), and energy-dispersive X-ray spectroscopy (EDS). The results are shown in Figures S3, S4 and Figure 3. SEM images (Figure S3) show that MoS_2_ nanosheets are loaded on the surface of diatomite in a three-dimensional flocculent structure. TEM and EDS further confirm that the crystal plane spacing of MoS_2_ (002) is 0.626 nm and the distribution of related elements (Figure S4). As depicted in Figure 3a,b, the DE/TiO_2_/MoS_2_/NCNF material primarily consists of a TiO_2_/N-doped carbon fiber matrix. Cylindrical diatomite particles are embedded within the three-dimensional network, with flocculent MoS_2_ dispersed on the fiber surfaces and surrounding areas. Figure 3c,d show HR-TEM images. In region ① of Figure 3c, lattice spacings of 0.249 nm correspond to the (101) plane of rutile TiO_2_, while in region ②, spacings of 0.351 nm correspond to the (101) plane of anatase TiO_2_, consistent with the XRD results. Lattice fringes of 0.626 nm, corresponding to the (002) plane of MoS_2_ [38], were observed in Figure 3d. Elemental mapping analysis (EDS, Figure 3f–i) confirmed the presence and homogeneous distribution of C, Mo, Ti, Si, O, and N elements on the sample surface, further corroborating the above analyses.

### 3.3. Electrochemical Performance Analysis

**Figure 4:**
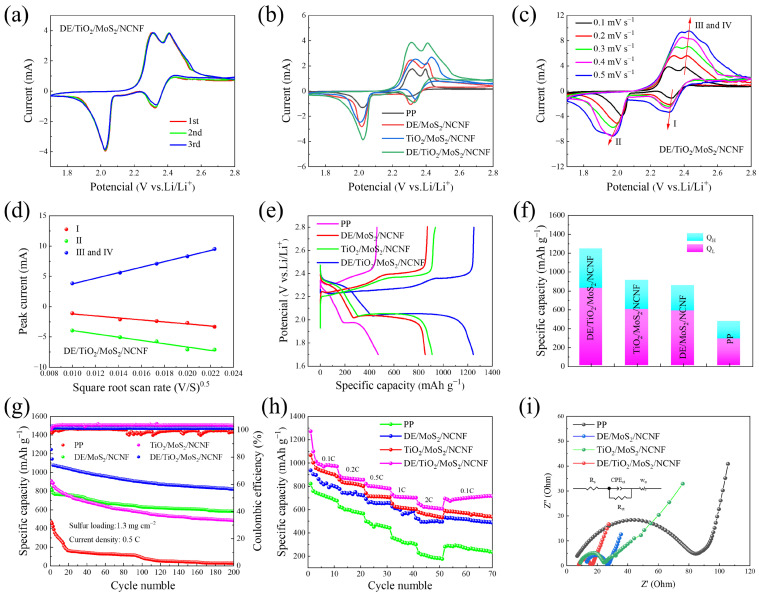
(**a**) CV test of cells employing the DE/TiO_2_/MoS_2_/NCNF interlayer at 0.1 mV s^−1^ sweep speed. (**b**) CV curves of batteries with different separators at 0.1 mV s^−1^ sweep speed. (**c**) CV test of cells with DE/TiO_2_/MoS_2_/NCNF-coated separators at different sweep speeds. (**d**) Relationship between peak current and the square root of scanning rate of CV curves of cells employing the DE/TiO_2_/MoS_2_/NCNF interlayer. Electrochemical performance test of separator with blank PP, DE/MoS_2_/NCNF, DE/TiO_2_/MoS_2_/NCNF, and TiO_2_/MoS_2_/NCNF coatings: (**e**) 0.5 C first-turn charge and discharge curve. (**f**) Capacity contributions of high-order polysulfide conversion (Q_H_) and low-order polysulfide conversion (Q_L_) and corresponding Q_L_/Q_H_ ratios. (**g**) Long-cycle tests. (**h**) Rate capability of batteries with different interlayers at different rates. (**i**) EIS spectra of batteries with different coated separators.

Cyclic voltammetry (CV) was performed within a voltage window of 1.7–2.8 V at a scan rate of 0.1 mV s^−1^ to understand the reaction processes during LiPS conversion (Figure S5a–c and Figure 4a). All CV curves exhibited a single oxidation peak (~2.41 V) and two reduction peaks (~2.08 V and ~2.32 V), corresponding to the oxidation and reduction reactions of sulfur species, explained as follows: reduction of elemental sulfur (S_8_) to long-chain Li_2_S_n_ (4 ≤ *n* ≤ 8), followed by its conversion to short-chain Li_2_S, and oxidation of Li_2_S back to S_8_ (reaction: 8Li_2_S → 16Li^+^ + S_8_) [39,40]. The DE/TiO_2_/MoS_2_/NCNF-coated cell exhibited the highest redox peak intensities, indicating superior lithium-ion diffusion kinetics. Furthermore, its CV curves showed high overlap after multiple cycles, reflecting excellent electrochemical reversibility and cycling stability.

Notably, at 0.1 mV s^−1^ (Figure 4b), the DE/TiO_2_/MoS_2_/NCNF-coated cell exhibited a negative shift in the oxidation peak potential and a positive shift in the reduction peak potential compared to other cells, accompanied by a significant increase in peak area. This indicates improved LiPS redox reaction kinetics and superior LiPS capture capability.

CV tests at various scan rates (0.1–0.5 mV s^−1^, Figure 4c and Figure S5d–f) were conducted to analyze reaction kinetics. As the scan rate increased (Figure 4a,b), the potential difference between oxidation and reduction peaks and the peak current density difference increased for all systems due to enhanced electrode polarization. The DE/TiO_2_/MoS_2_/NCNF cell consistently showed an oxidation peak shifting towards lower potentials at all scan rates. This enhanced electrocatalytic activity is attributed to the synergistic interfacial effect between TiO_2_ nanoparticles and MoS_2_ nanosheets in the composite, effectively lowering the energy barrier for Li_2_S oxidation. The CV curves of the DE/TiO_2_/MoS_2_/NCNF cell also exhibited significantly larger peak areas.

The Li^+^ diffusion coefficient ($D_{{Li}^{+}}$), a key factor influencing reaction kinetics, was determined from the CV curves obtained at different scan rates (0.1–0.5 mV s^−1^, Figure 4c). We combined the peak current and the Randles–Sevcik equation [41] as follows:

$$I_{p}\;=\;2.69\;\times {\;10}^{5}n^{1.5}AD_{{Li}^{+}}^{0.5}v^{0.5}C_{{Li}^{+}}$$

where $I_{p}$ is the peak current, n is the number of electrons involved in the redox reaction, A is the cathode area (1.13 cm^2^ in this case), $D_{{Li}^{+}}$ is the lithium-ion diffusion coefficient, $C_{{Li}^{+}}$ is the lithium-ion concentration, and v is the scan rate. The linear relationship between peak current ($I_{p}$) and the square root of the scan rate ($v^{0.5}$) (slope proportional to $D_{{Li}^{+}}^{0.5}$) is shown in Figure 4d and Figure S5g–i. The DE/TiO_2_/MoS_2_/NCNF system exhibited significantly steeper slopes at reduction peak I (Li_2_Sn formation), reduction peak II (Li_2_S formation), and oxidation peak III (S_8_ regeneration). $D_{{Li}^{+}}$ values calculated using the Randles–Sevcik equation (Table 1) confirmed that the DE/TiO_2_/MoS_2_/NCNF-coated cell possessed the highest Li^+^ diffusion coefficients. This indicates the following significant differences in electrode reaction kinetics: the synergistic TiO_2_/MoS_2_ interface combined with the 3D porous structure of diatomite effectively shortened the Li^+^ diffusion path at the electrode/electrolyte interface.

At a rate of 0.5 C, the first-week discharge capacity of the DE/MoS_2_-coated separator battery reached 1334.5 mAh g^−1^, and the capacity retention rate after 200 cycles was 50.5%, which was significantly better than that of the single-component modified layer (Figure S6c). Li-S batteries were assembled using different composite-coated separators, namely DE/MoS_2_/NCNF, DE/TiO_2_/MoS_2_/NCNF, TiO_2_/MoS_2_/NCNF, and a pristine PP separator. Initial charge–discharge curves at 0.5 C are shown in Figure 4e. All modified batteries exhibited typical dual discharge plateaus. The DE/TiO_2_/MoS_2_/NCNF-coated cell displayed the smallest polarization potential difference (ΔE), primarily attributed to the hierarchical porous structure of diatomite enhancing physical adsorption and the synergistic interface between TiO_2_ nanoparticles and MoS_2_ nanosheets providing abundant Lewis acidic sites, thus effectively accelerating LiPS conversion kinetics. Analysis of LiPS conversion efficiency (Figure 4f) revealed that the DE/TiO_2_/MoS_2_/NCNF system achieved the highest values for both Q_H_ (high-order LiPS conversion) and Q_L_ (low-order LiPS conversion), indicating that the composite not only strengthened LiPS capture but also promoted their deep conversion to solid Li_2_S [42].

The cycling stability of cells with different separators (pristine PP, DE/MoS_2_/NCNF, DE/TiO_2_/MoS_2_/NCNF, and TiO_2_/MoS_2_/NCNF) at 0.5 C is shown in Figure 4g. The initial discharge specific capacity of the DE/TiO2/MoS2/NCNF-coated cell was 1245.6 mAh g^−1^, significantly higher than those of the pristine PP (471.4 mAh g^−1^), DE/MoS_2_/NCNF (833.1 mAh g^−1^), and TiO_2_/MoS_2_/NCNF (909.9 mAh g^−1^) cells. After 200 cycles, the DE/TiO_2_/MoS_2_/NCNF-coated cell maintained a discharge specific capacity of 821.3 mAh g^−1^, corresponding to a capacity retention of 65.94%. To further verify the long-cycle performance of the composite material, after 1000 cycles at a current density of 0.5 C, the attenuation rate for each cycle was 0.068% (Figure S7). Furthermore, this paper integrates some of the research results on Li-S batteries from both domestic and international sources, as shown in Table S1. For example, compared with other coating materials, the Li-S batteries with DE/MoS_2_- and DE/TiO_2_/MoS_2_/NCNF-coated separators all demonstrated excellent battery cycling performance. Among them, the Li-S battery with the DE/TiO_2_/MoS_2_/NCNF separator achieved an extremely low capacity degradation rate of 0.17% during the battery cycling process, confirming that it can effectively improve the redox reactions of LiPSs and suppress the “shuttle effect” inside the battery.

Rate performance tests show that DE/MoS_2_ can still release 584.3 mAh g^−1^ at a high rate of 2 C (Figure S6d). Rate capability was evaluated at various rates (0.1 C, 0.2 C, 0.5 C, 1 C, and 2 C), as shown in Figure 4h. The DE/TiO_2_/MoS_2_/NCNF-coated cell delivered discharge specific capacities of 1272.9 mAh g^−1^ at 0.1 C, 888.8 mAh g^−1^ at 0.2 C, 821.8 mAh g^−1^ at 0.5 C, 730.6 mAh g^−1^ at 1 C, and 651.4 mAh g^−1^ at 2 C, outperforming cells with other separators at corresponding rates. The discharge capacity gradually decreased with increasing current density due to kinetic overpotential and polarization. When the rate returned to 0.1 C, the DE/TiO_2_/MoS_2_/NCNF-coated cell recovered a capacity of 595.6 mAh g^−1^. The porous structure of diatomite, the strong polarity of MoS_2_, and the enhanced overall conductivity provided by the TiO_2_/carbon fiber network, collectively offering abundant active sites for LiPS adsorption, effectively suppressed the shuttle effect and ensured superior rate performance.

Electrochemical impedance spectroscopy (EIS) Nyquist plots (Figure 4i) and equivalent circuit fitting were used to understand charge transfer kinetics. R_s_, R_ct_, and W_o_ represent the ohmic resistance, charge transfer resistance, and Warburg impedance for solid-phase diffusion, respectively. EIS analysis indicates that its charge transfer resistance (R_ct_) is the smallest (Figure S8), suggesting that the synergic effect of DE and MoS_2_ effectively enhances LiPS conversion kinetics. The charge transfer impedances of the PP separator, DE/MoS_2_/NCNF, and TiO_2_/MoS_2_/NCNF are 79.70 Ω, 13.61 Ω, and 15.39 Ω, respectively. The DE/TiO_2_/MoS_2_/NCNF-coated separator exhibited the smallest semicircle diameter, indicating the lowest R_ct_ (7.65 Ω). Fitted resistance values confirmed that DE/TiO_2_/MoS_2_/NCNF consistently yielded the lowest R_ct_, implying reduced electrochemical impedance, faster charge transfer, and accelerated reaction kinetics compared to other separators.

## 4. Conclusions

Building upon previous research, this study successfully constructed a hierarchically structured DE/TiO_2_/MoS_2_/NCNF composite functional material via an innovative process combining electrospinning and high-temperature carbonization. This composite was applied as a separator coating in Li-S batteries. The material primarily consists of a TiO_2_/N-doped carbon fiber matrix, with cylindrical/disk-shaped diatomite particles embedded within it and flocculent MoS_2_ dispersed on the fiber surfaces and surroundings. The composite was thoroughly characterized using XRD, FT-IR, and XPS. Electrochemical testing demonstrated that the Li-S battery with the DE/TiO_2_/MoS_2_/NCNF-coated separator delivered an initial discharge specific capacity of 1245.6 mAh g^−1^ at 0.5 C. After 200 cycles, a discharge specific capacity of 821.3 mAh g^−1^ was retained, corresponding to a capacity retention of 65.94%, alongside an excellent rate capability. Furthermore, CV analysis revealed that the DE/TiO_2_/MoS_2_/NCNF-coated cell exhibited a negative shift in the oxidation peak potential, a positive shift in the reduction peak potential, and increased peak area, indicating effectively shortened Li^+^ diffusion paths and accelerated Li^+^ migration rates within the battery. EIS analysis and equivalent circuit fitting confirmed that the DE/TiO_2_/MoS_2_/NCNF separator effectively minimized charge transfer resistance (R_ct_). These comprehensive analyses demonstrate that when used as a functional separator coating, the DE/TiO_2_/MoS_2_/NCNF quaternary composite significantly enhances the electrochemical performance of Li-S batteries.

## Figures and Tables

**Table 1:** Lithium-ion diffusion coefficients of membrane batteries with different coatings.

Materials	I (cm^2^ s^−1^)	II (cm^2^ s^−1^)	III and IV (cm^2^ s^−1^)
PP	(1.24 ± 0.01) × 10^−10^	(4.71 ± 0.02) × 10^−10^	(5.69 ± 0.05) × 10^−10^
DE/MoS_2_/NCNF	(1.38 ± 0.01) × 10^−9^	(3.05 ± 0.05) × 10^−10^	(8.35 ± 1.31) × 10^−10^
TiO_2_/MoS_2_/NCNF	(1.25 ± 0.001) × 10^−9^	(2.33 ± 0.02) × 10^−10^	(5.96 ± 0.04) × 10^−10^
DE/TiO_2_/MoS_2_/NCNF	(2.85 ± 0.001) × 10^−9^	(3.71 ± 0.04) × 10^−10^	(1.01 ± 0.01) × 10^−9^

## Data Availability

The original contributions presented in this study are included in the article/Supplementary Material. Further inquiries can be directed to the corresponding author.

## Supplementary Materials

The following supporting information can be downloaded at: https://www.mdpi.com/article/10.3390/ma18153654/s1, Figure S1: (a) XRD pattern of DE, MoS_2_, DE/MoS_2_ material; (b) FT-IR diagram of DE, DE/MoS_2_. References [43,44,45] are cited in the supplementary materials. Figure S2: The XPS survey spectrum of (a) DE/MoS_2_; High-resolution XPS spectrum of (b–d) C 1s, Mo 3d, a nd S 2p. References [46,47] are cited in the supplementary materials. Figure S3: SEM images in DE/MoS_2_ of (a,b) diatomite and (c,d) MoS_2_. Figure S4: (a) HR-TEM diagram, (b) SAED diagram, and (c,d) Elemental mapping of DE/MoS_2_ materials. Figure S5: CV test of cells with different material coated separators at 0.1 mV s^−1^ sweep speed: (a) DE/MoS_2_/NCNF; (b) TiO_2_/MoS_2_/NCNF; (c) PP. CV test of cells with different material coated membranes and blank PP membranes at different sweep speeds: (d) DE/MoS_2_/NCNF; (e) TiO_2_/MoS_2_/NCNF; (f) PP. Relationship between peak current and the square root of scanning rate of CV curves of cells with different coating materials: (g) DE/MoS_2_/NCNF; (h) TiO_2_/MoS_2_/NCNF; (i) PP. Figure S6: Electrochemical performance test of Li-S batteries with different separators: (a) First cycle charge and discharge curve of blank PP, DE, MoS_2_, DE/MoS_2_; (b) Blank PP, DE, MoS_2_, DE/MoS_2_ discharge platform capacity; (c,d) 0.2 C long cycle test and rate performance test of blank PP, DE, MoS_2_, DE/MoS_2_. Figure S7: The long-cycle performance of DE/TiO_2_/MoS_2_/NCNF. Figure S8: EIS of separator batteries with different coating materials. Table S1: Comparison of electrochemical performance of this work with previous work. References [48,49,50,51,52,53,54] are cited in the supplementary materials.
