# Supplementary material for: The Design of Diatomite/TiO2/MoS2/Nitrogen-Doped Carbon Nanofiber Composite Separators for Lithium–Sulfur Batteries
Source: Materials (Basel). 2025 Aug 4;18(15):3654. doi: 10.3390/ma18153654 (PMC12348443; doi:10.3390/ma18153654)
Supplement: Supplementary file 1 [file materials-18-03654-s001.zip › materials-3765906-supplementary.pdf]

materials

MDPI

Supporting information

The Design of Diatomite/TiO_2/MoS_2/Nitrogen-Doped Carbon Nanofiber Composite Separators for Lithium–Sulfur Batteries

Wei Zhong [1], Wenjie Xiao [2], Jianfei Liu [2], Chuxiao Yang [3], Sainan Liu [2] and Zhenyang Cai [1,*]

1 School of Materials Science and Engineering, Central South University, Changsha 410083, China; zhong9340@gmail.com
2 School of Minerals Processing and Bioengineering, Central South University, Changsha 410083, China; 225612081@csu.edu.cn (W.X.); csu245601012@csu.edu.cn (J.L.); lsn@csu.edu.cn (S.L.)
3 School of Business, Central South University, Changsha 410083, China; lucas04031@163.com
* Correspondence: csuczy@csu.edu.cn

Results and Discussion

The different crystalline phase structures of the materials were analyzed using X-ray diffraction (XRD). Figure S1a shows the XRD patterns of DE, MoS_2, and the DE/MoS_2 composite. Characteristic peaks of amorphous SiO_2[43], specifically a broad peak at $2\theta = 21.8°$, were observed in both the pure diatomite (DE) and the DE/MoS_2 composite powder. Peaks at 20.84° and 26.62° correspond to the (110) and (011) planes of silica (PDF#01-075-8321). Distinct diffraction peaks at 14.13°, 32.91°, 35.97°, and 58.76° were unambiguously assigned to the (002), (100), (102), and (110) planes of molybdenum disulfide (MoS_2)[44]. These spectral data confirm the coexistence of both diatomite (DE) and MoS_2 phases in the prepared DE/MoS_2 composite, indicating that both components retained their intrinsic crystalline structural features within the composite material.

Figure S1b displays the Fourier transform infrared (FTIR) spectra of DE and DE/MoS_2. In the infrared absorption spectra, a broad peak near 3700–3050 cm^{-1}, corresponding to the stretching vibration of Si-OH groups, indicates the presence of abundant silanol groups on the diatomite surface. The absorption peak at 1632 cm^{-1} is attributed to the stretching vibration of water hydroxyl groups (-OH). Peaks around 1100, 795, and 467 cm^{-1} correspond to the transverse and longitudinal symmetric stretching vibrations and the bending vibration of the Si-O-Si bond, respectively. In the FTIR spectrum of DE/MoS_2, peaks at 1049 cm^{-1} and 879 cm^{-1} are assigned to the bending vibrations of S-Mo-S, while the peak at 428 cm^{-1} corresponds to the Mo-S stretching vibration[45]. The characteristic absorption peaks in other positions largely overlap with those of pure diatomite, demonstrating that the DE/MoS_2 composite retained the fundamental functional group structures of both diatomite and MoS_2.

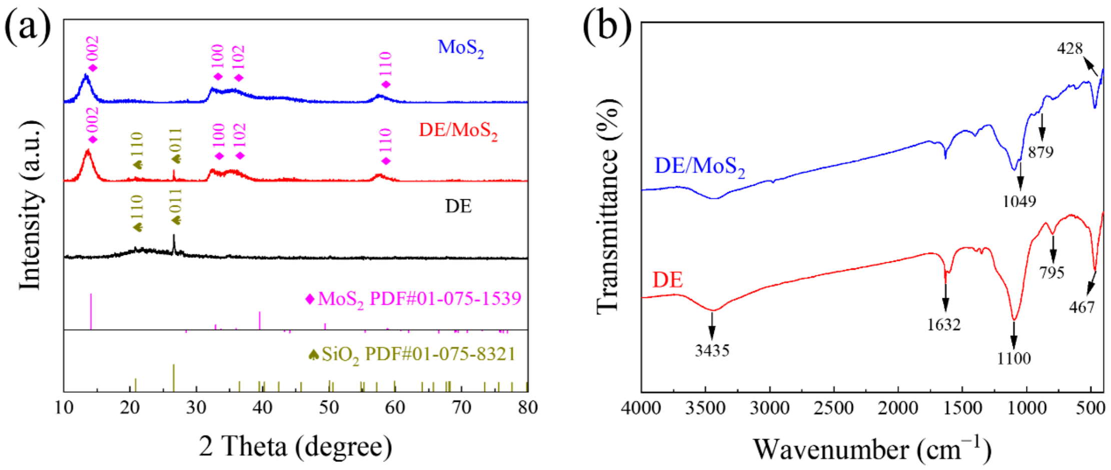

Figure S1. (**a**) XRD pattern of DE, MoS_2, DE/MoS_2 material; (**b**) FT-IR diagram of DE, DE/MoS_2.

X-ray photoelectron spectroscopy (XPS) was systematically employed to analyze the surface elemental composition and chemical valence states of the prepared materials. The corresponding spectra are shown in Figure S2. Peaks in the full survey scan (0–1200 eV binding energy) confirmed the presence of O, C, Mo, S, and Si elements, with peak heights reflecting relative elemental abundances. Data were calibrated using the standard C 1s peak at 284.8 eV. The high-resolution C 1s spectrum (Figure S2b) was deconvoluted into four peaks: peaks at 284.8 eV and 286.3 eV are assigned to C-C and C-O bonds, respectively. The high-resolution Mo 3d spectrum (Figure S2c) was deconvoluted into three peaks: peaks at 231.9 eV (Mo 3d3/2) and 228.6 eV (Mo 3d5/2), along with the S 2s peak at 225.8 eV, confirm the presence of Mo in the +4 oxidation state within the composite[46]. The high-resolution S 2p spectrum (Figure S2d) was deconvoluted into two peaks at 161.5 eV (S 2p3/2) and 162.8 eV (S 2p1/2)[47]. Collectively, this analysis confirms the successful preparation of the DE/MoS_2 composite.

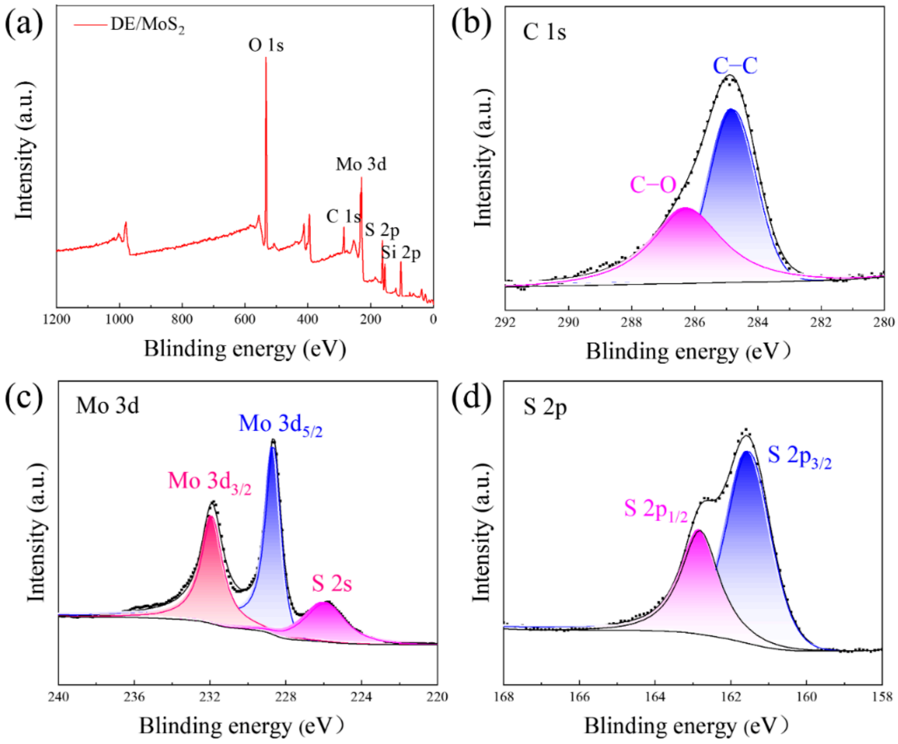

Figure S2. The XPS survey spectrum of (**a**) DE/MoS_2; High-resolution XPS spectrum of (**b**–**d**) C 1s, Mo 3d, and S 2p.

The structural morphology of the materials was observed using scanning electron microscopy (SEM). Figure S3 shows SEM images of the DE/MoS_2 composite. Figures S3(a, b) reveal the morphology of diatomite within the composite, displaying porous cylindrical and disk-shaped structures, with disk diameters around 35 μm. Flocculent MoS_2 is attached to the surface and surrounding areas of the diatomite particles. Figures S3(b, d) present the MoS_2 microstructure at different magnifications. It can be observed that MoS_2 nanosheets self-assemble into a three-dimensional flocculent network structure. This open layered architecture significantly increases the material/electrolyte contact interface, facilitating rapid lithium-ion migration within the battery.

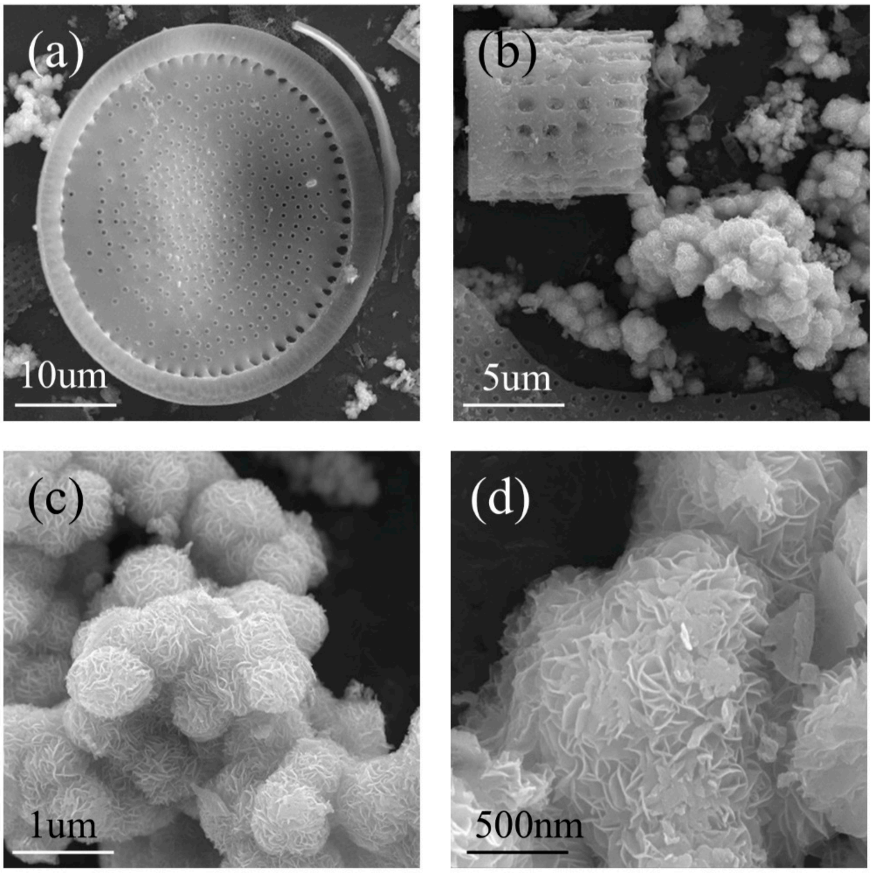

Figure S3. SEM images in DE/MoS_2 of (**a**–**b**) diatomite and (**c**–**d**) MoS_2.

Further microstructural analysis of the prepared materials was conducted using transmission electron microscopy (TEM). Figure S4 presents TEM images of DE/MoS_2, the corresponding selected area electron diffraction (SAED) pattern, and the EDS spectrum. Figure S4a) shows a local high-resolution TEM (HR-TEM) image of DE/MoS_2, where lattice fringes of 0.626 nm, corresponding to the (002) plane of MoS_2, are visible. The SAED pattern of DE/MoS_2 (Figure S4b) displays concentric rings of different radii, indicating that the composite material is polycrystalline. This finding is consistent with the XRD results. EDS elemental mapping of the material surface (Figures S4(c, d)) confirmed the presence of C, O, Mo, and S elements, demonstrating the successful synthesis of the MoS_2 material.

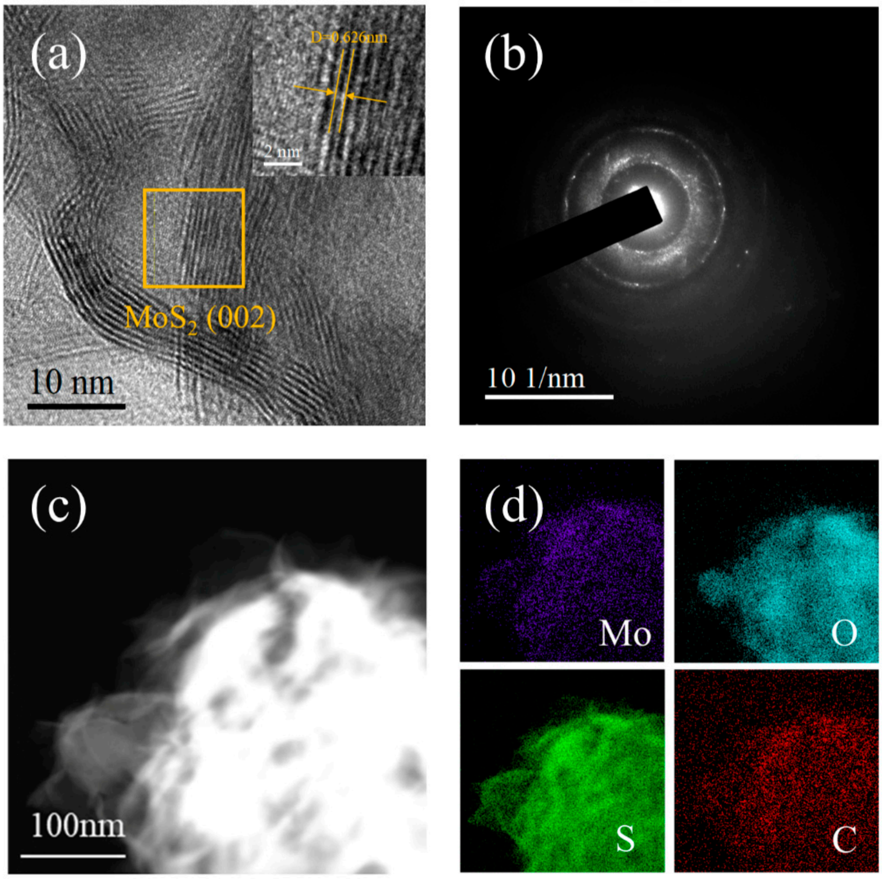

Figure S4. (**a**) HR-TEM diagram, (**b**) SAED diagram, and (**c–d**) Elemental mapping of DE/MoS_2 materials.

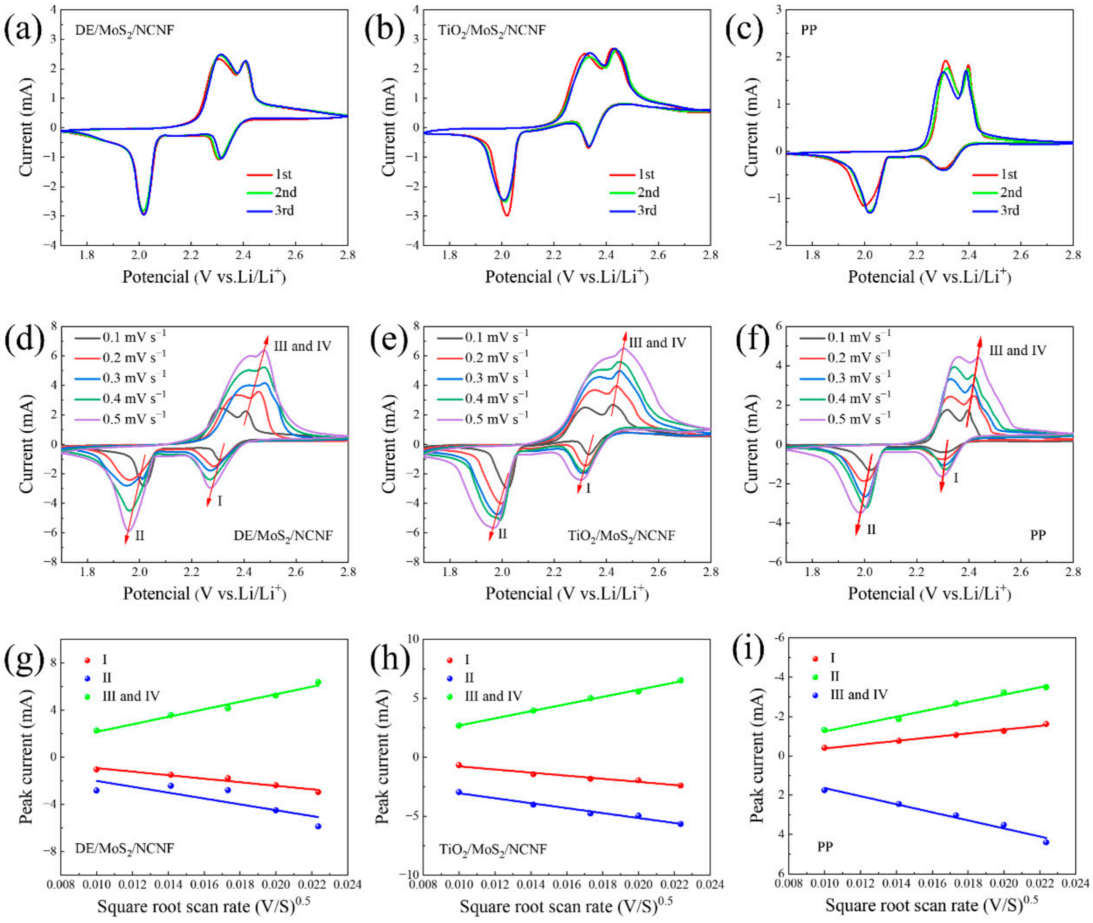

Figure S5. CV test of cells with different material coated separators at 0.1 mV s^{-1} sweep speed: (**a**) DE/MoS_2/NCNF; (b) TiO_2/MoS_2/NCNF; (**c**) PP. CV test of cells with different material coated membranes and blank PP membranes at different sweep speeds: (**d**) DE/MoS_2/NCNF; (**e**) TiO_2/MoS_2/NCNF; (**f**) PP. Relationship between peak current and the square root of scanning rate of CV curves of cells with different coating materials: (**g**) DE/MoS_2/NCNF; (h) TiO_2/MoS_2/NCNF; (i) PP.

To systematically evaluate the impact of different modification layers on the electrochemical performance of Li-S batteries, pristine PP separators and modified separators coated with DE, MoS_2, and the DE/MoS_2 composite were applied in Li-S batteries. Polarization characteristics were primarily investigated via galvanostatic charge–discharge testing. The initial charge–discharge curves obtained at a rate of 0.5 C are shown in Figure S5a. All modified cells exhibited the characteristic dual discharge voltage plateaus. The cell with the DE/MoS_2 composite coating demonstrated the most favorable electrochemical polarization behavior, with a plateau potential difference (ΔE) of only 0.373 V, significantly lower than that of the cell with the pristine PP separator. This phenomenon may be attributed to the synergistic mechanism of the DE/MoS_2 composite structure: on the one hand, the three-dimensional porous structure of diatomite enhances the anchoring capability for LiPSs through physical adsorption; on the other hand, the uniformly distributed MoS_2 nanosheets expose abundant edge active sites, whose Lewis acidic surfaces effectively promote the liquid-to-solid conversion kinetics of LiPSs. Figure S5b displays the high-order LiPSs conversion (Q_H), low-order LiPSs conversion (Q_L), and the corresponding Q_L/Q_H ratio for the different separator materials (DE, MoS_2, DE/MoS_2). Compared to separators modified with DE or MoS_2 alone, the DE/MoS_2 composite coating exhibited superior electrochemical characteristics: at 0.5 C, it achieved the highest capacities for both the high- and low-voltage plateaus (Q_H and Q_L) in the initial discharge curve, and the plateau potential difference (ΔE) was reduced to 0.373 V. Compared to single-component coatings, the composite structure, leveraging the synergistic effect of physical adsorption by diatomite and chemical catalysis by MoS_2, not only effectively reduced the nucleation barrier for Li_2S but also significantly improved the utilization of active material.

To further verify the cycling stability advantage of the composite coating, long-term discharge performance tests were conducted on the four types of cells at a current density of 0.5 C, as shown in Figure S5c. Electrochemical performance results show that the Li-S battery equipped with the DE/MoS_2 functionalized separator achieved an initial discharge specific capacity of 1334.5 mAh g^{-1}. This represents improvements of 183.1%, 95.6%, and 5.2% compared to batteries with the pristine PP separator (471.4 mAh g^{-1}), pure DE-modified separator (682.3 mAh g^{-1}), and single-component MoS_2-coated separator (1268.8 mAh g^{-1}), respectively. During long-term cycling, the composite battery maintained a reversible capacity of 674.5 mAh g^{-1} after 200 charge–discharge cycles, corresponding to a capacity retention of 50.5%, significantly outperforming the other control groups. Rate performance was evaluated for cells with PP, MoS_2, and DE/MoS_2 coated separators. Prior to electrochemical measurements, coin cells were rested overnight and activated by a single charge/discharge cycle at 0.05 C (where 1.0 C = 1675 mA g^{-1}). Electrochemical test results (Figure S5d) demonstrate that the Li-S battery with the DE/MoS_2 functionalized separator exhibited excellent electrochemical performance across various current densities. At a low rate of 0.1 C, the initial discharge capacity reached 1137.8 mAh g^{-1}. As the current density was stepwise increased to 0.2 C, 0.5 C, 1 C, and 2 C, the initial discharge specific capacities were 863.2 mAh g^{-1}, 757.2 mAh g^{-1}, 708.1 mAh g^{-1}, and 584.3 mAh g^{-1}, respectively, far exceeding those of batteries with pristine PP, DE, or MoS_2 coated separators. At all tested rates, the discharge capacity of the cell modified with the DE/MoS_2 separator was significantly superior to those with pristine PP, pure DE, or single-component MoS_2 coatings. Discharge capacity gradually decreased with increasing current density due to kinetic overpotential and polarization. When the current density was reset to 0.1 C, the reversible discharge capacity of the Li-S battery with the DE/MoS_2 functionalized separator recovered to 661.2 mAh g^{-1}, corresponding to a capacity retention rate of 58.11%. The loading and adsorption capacity of diatomite provide a natural porous network that not only serves as a uniform dispersion carrier for MoS_2 nanosheets but also improves the wettability between the active material and the electrolyte. Concurrently, the edge active sites of MoS_2 enhance LiPSs conversion kinetics through chemical adsorption. The synergistic action of these components improves the battery's cycling stability.

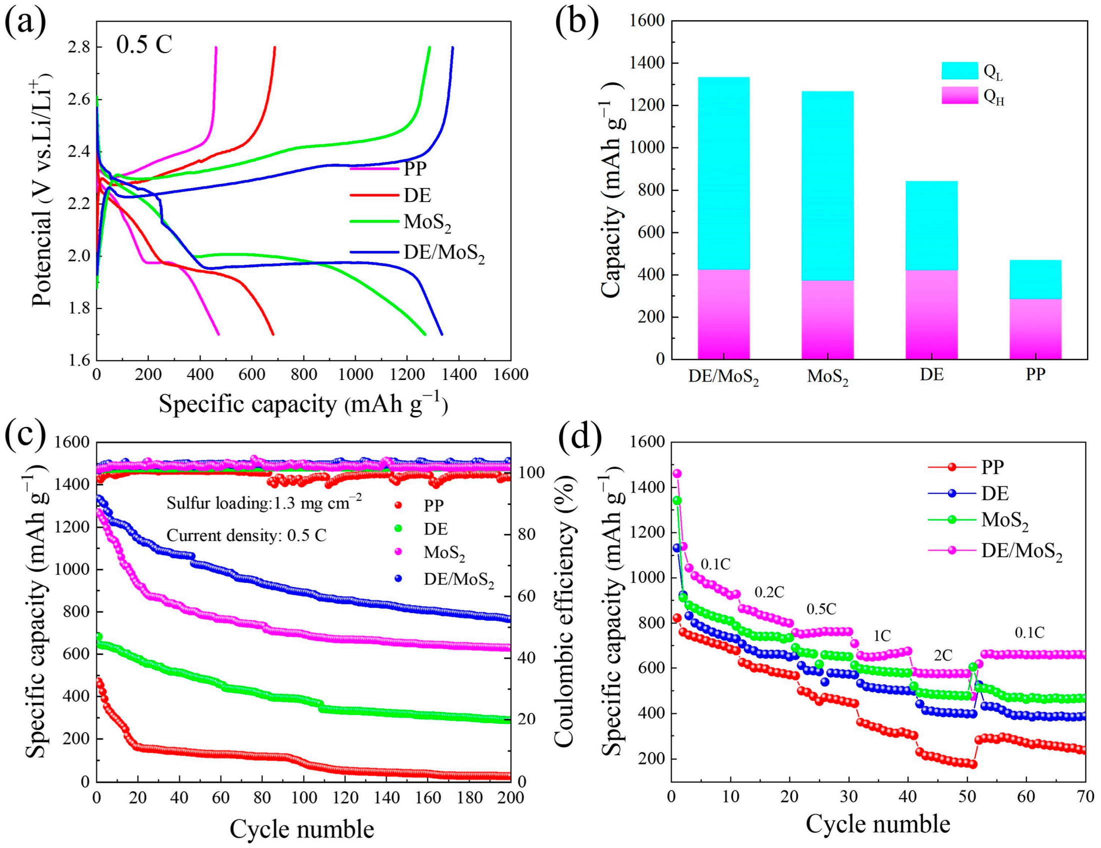

Figure S6. Electrochemical performance test of Li-S batteries with different separators: (**a**) First.cycle charge and discharge curve of blank PP, DE, MoS_2, DE/MoS_2; (**b**) Blank PP, DE, MoS_2, DE/MoS_2 discharge platform capacity; (**c**–**d**) 0.2 C long cycle test and rate performance test of blank PP, DE, MoS_2, DE/MoS_2.

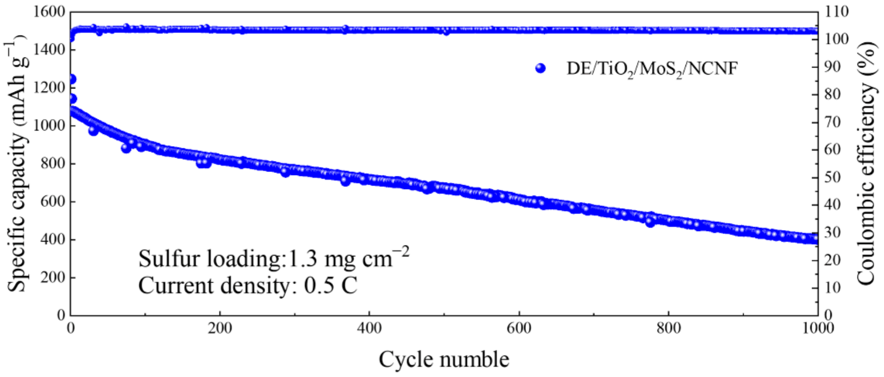

Figure S7. The long-cycle performance of DE/TiO_2/MoS_2/NCNF.

Electrochemical impedance spectroscopy (EIS) was utilized to probe the charge transfer kinetics within the batteries. EIS measurements were performed on cells with DE, MoS_2, DE/MoS_2, and pristine PP separators. The resulting Nyquist plots are shown in FigureS8, along with the corresponding equivalent circuit. The EIS analysis results indicate that the DE/MoS_2 composite-coated separator exhibited the most favorable ion/electron transport characteristics. As shown in the inset of Figure S8 and Table S1, equivalent circuit fitting data revealed that this system possessed the smallest interfacial charge transfer resistance (R_{ct}). This suggests that DE/MoS_2 significantly accelerates the redox reaction kinetics of LiPSs, thereby reducing the overall battery impedance and enhancing the charge transfer rate within the cell.

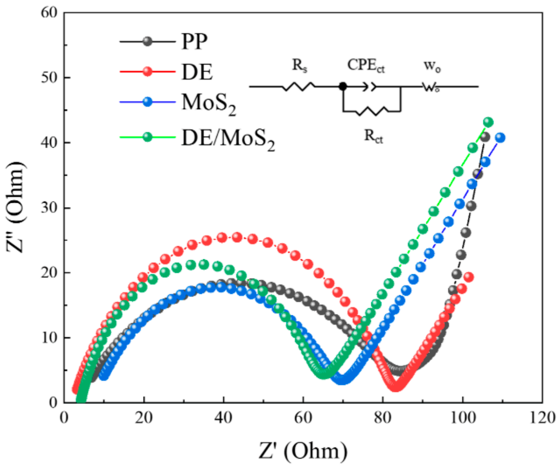

Figure S8. EIS of separator batteries with different coating materials.

Table S1. Comparison of electrochemical performance of this work with previous work.

Cell	Current density (1 C=1675 mA g^{-1})	Cycle number	Initial capacity ($mAh\ g^{-1}$)	Capacity decay ratio(%)	*Ref.*
EHNT	0.5 C	150	1090.1	0.23	[48]
MWCNT/2D-MoS_2	0.5 C	100	1165	0.14	[49]
S-ANF	0.5 C	200	1108	0.24	[50]
MoS_2@CoS_2	0.2 C	100	1100	0.18	[51]
TiO_2NT/RGO	0.2 C	100	1303	0.52	[52]
MC/TO	0.1 C	100	1369	0.24	[53]
CTC	0.5 C	100	990	0.38	[54]
DE/MoS_2	0.5 C	200	1334	0.25	This work
NCNF/TiO_2/DE-800	0.5 C	200	1245.6	0.17	This work

References

43. Sun, Z.;Zheng, S.;Ayoko, G.A.;Frost, R.L.;Xi, Y. Degradation of simazine from aqueous solutions by diatomite-supported nanosized zero-valent iron composite materials. *JOURNAL OF HAZARDOUS MATERIALS* **2013**, 263, 768-777. https://doi.org/10.1016/j.jhazmat.2013.10.045.
44. Zheng, F.H.;Pan, Q.C.;Yang, C.H.;Xiong, X.H.;Ou, X.;Hu, R.Z.;Chen, Y.;Liu, M.L. Sn-MoS_2-C@C Microspheres as a Sodium-Ion Battery Anode Material with High Capacity and Long Cycle Life. *CHEMISTRY-A EUROPEAN JOURNAL* **2017**, 23, 5051-5058. https://doi.org/10.1002/chem.201605005.
45. Zhang, H.;Zeng, L.;Wu, X.;Lian, L.;Wei, M. Synthesis of MoO_2 nanosheets by an ionic liquid route and its electrochemical properties. *J. Alloy. Compd.* **2013**, 580, 358-362. https://doi.org/https://doi.org/10.1016/j.jallcom.2013.06.100.
46. Yu, X.Y.;Feng, Y.;Jeon, Y.;Guan, B.;Lou, X.W.;Paik, U. Formation of Ni-Co-MoS_2 Nanoboxes with Enhanced Electrocatalytic Activity for Hydrogen Evolution. *ADVANCED MATERIALS* **2016**, 28, 9006-9011. https://doi.org/10.1002/adma.201601188.
47. Nde, D.T.;Vadapalli, H.;Roy, N.;Venkatesan, R.;Li, X.;El-Marghany, A.;Arla, S.K.;Boya, V.K.N.;Joo, S.W. Flower-like microstructures of Co@MoS_2@N-RGO: A promising platform for solar-driven water splitting through engineering 1T phase MoS_2 and N-RGO integration. *Electrochim. Acta* **2025**, 525, 146115. https://doi.org/https://doi.org/10.1016/j.electacta.2025.146115.
48. Sun, L.;Li, J.;Li, Z.;Li, W.;Lv, G.;Liao, L. Tubular clay of halloysites as separator modification layers enabling effective polysulfide fixing and robust Li-S batteries. *Applied Materials Today* **2025**, 44, 102677. https://doi.org/https://doi-org-s-154.libdb.csu.edu.cn/10.1016/j.apmt.2025.102677.
49. Pundir, A.C.;Sil, A. Synergetic effect of 2D-MoS_2 nanoflakes functionalised separator supported by hierarchical porous carbon/sulfur nanoparticle composite cathode for improved polysulfide conversion in Li-S battery. *J. Energy Storage* **2025**, 112, 115594. https://doi.org/https://doi-org-s-154.libdb.csu.edu.cn/10.1016/j.est.2025.115594.
50. He, J.;Li, W.;Pang, R.;Lu, P.;Zhang, M.;Feng, R.;Yang, B. Regulating pore structure of aramid nanofiber (ANF) separators for lithium–sulfur (Li–S) batteries. *Materials Today Energy* **2024**, 44, 101640. https://doi.org/https://doi-org-s-154.libdb.csu.edu.cn/10.1016/j.mtener.2024.101640.
51. Tu, C.;Zhang, Z.;Qi, X.;Wang, F.;Yang, Z. Heteroelectrocatalyst MoS_2@CoS_2 modified separator for Li-S battery: Unveiling superior polysulfides conversion and reaction kinetics. *Chem. Eng. J.* **2024**, 499, 155915. https://doi.org/https://doi-org-s-154.libdb.csu.edu.cn/10.1016/j.cej.2024.155915.
52. Gui, Y.;Chen, P.;Liu, D.;Fan, Y.;Zhou, J.;Zhao, J.;Liu, H.;Guo, X.;Liu, W.;Cheng, Y. TiO_2 nanotube/RGO modified separator as an effective polysulfide-barrier for high electrochemical performance Li-S batteries. *J. Alloy. Compd.* **2022**, 895, 162495. https://doi.org/https://doi-org-s-154.libdb.csu.edu.cn/10.1016/j.jallcom.2021.162495.
53. Liu, S.;Li, C.;Liu, D. Modified Separator Based on mesoporous carbon/TiO_2 composites as Advanced Polysulfide Adsorber for High Electrochemical Performance Li-S Batteries. *J. Alloy. Compd.* **2021**, 862, 158381. https://doi.org/https://doi-org-s-154.libdb.csu.edu.cn/10.1016/j.jallcom.2020.158381.
54. Yang, Y.;Xu, H.;Wang, S.;Deng, Y.;Qin, X.;Qin, X.;Chen, G. N-doped carbon-coated hollow carbon nanofibers with interspersed TiO_2 for integrated separator of Li-S batteries. *Electrochim. Acta* **2019**, 297, 641-649. https://doi.org/https://doi-org-s-154.libdb.csu.edu.cn/10.1016/j.electacta.2018.12.009.
